# Near-Complete Genome Sequence of a Potential Foot-and-Mouth Disease Virus Serotype A Vaccine Strain Isolated from Bangladesh

**DOI:** 10.1128/MRA.00031-19

**Published:** 2019-09-12

**Authors:** M. Al Amin, M. Rahmat Ali, A. S. M. Rubayet Ul Alam, Mohammad Anwar Siddique, Huzzat Ullah, Munawar Sultana, M. Anwar Hossain

**Affiliations:** a Department of Microbiology, University of Dhaka, Dhaka, Bangladesh; KU Leuven

## Abstract

The near-complete genome sequence of foot-and-mouth disease virus (FMDV) serotype A potential vaccine strain BAN/CH/Sa-304/2016 is reported here. Its genome revealed antigenic heterogeneity with the current Indian vaccine strain IND40/00, with four amino acid substitutions in antigenically critical sites of the VP1 protein.

## ANNOUNCEMENT

Foot-and-mouth disease (FMD) is a highly contagious disease of domestic and wild cloven-hoofed animals worldwide (1). Foot-and-mouth disease virus (FMDV), the etiological agent of the disease, is a positive-sense RNA virus of the genus Aphthovirus in the family *Picornaviridae*. FMDV exists as seven immunologically distinct serotypes (O, A, C, Asia1, and SAT1 to -3) with multiple topotypes, lineages, and sublineages (1, 2). Among all seven FMDV serotypes, serotype A is genetically and antigenically the most diverse and grouped into Asia, Europe-South America, and Africa topotypes (3). The disease is endemic in Bangladesh, with circulation of serotypes O, A, and Asia1 being found, of which serotype O is the most abundant circulatory virus, followed by serotypes A and Asia1 (2). All the previously reported type A viruses from Bangladesh belonged to genotype VII of the Asia topotype (4, 5).

Vaccination is the main tool for controlling FMD in areas of endemicity, but repeated vaccine failure in Bangladesh warrants an effective vaccine tailored to circulatory serotypes (4, 6). Therefore, we proposed FMDV serotype A isolate BAN/CH/Sa-304/2016 as a potential vaccine strain based on a two-dimensional microneutralization test (2D-MNT) and a strong antigenic relationship with contemporary serotype A strains of Bangladesh (*r* > 0.3) (7). The serotype A strain BAN/CH/Sa-304/2016 was isolated from tongue tissue of an infected cow, collected on 29 September 2016 from a military dairy farm in Chittagong, Bangladesh. The viral RNA was extracted from the cell culture supernatant at passage 3 in a BHK-21 cell line using a Maxwell 16 standard elution volume (SEV) RNA cartridge in an automated Maxwell 16-nucleic-acid extraction instrument, following the manufacturer’s protocol (Promega, USA). Extracted RNA was reverse transcribed using the GoScript reverse transcription system (Promega) with random and oligo(dT) primers, per the manufacturer’s instructions, to synthesize cDNA. From cDNA, a total of 20 overlapping amplicons spanning the viral genome were generated using the following 38 internal primers: A1F, A1R, A2F3, A2R, 682F, 5′ UTR-4F, 5′ UTR-4R, A4F, A4R, A5R, A6F, A6R, A7F, A7R, A8F2, A8R2, A9F, A9R, A10F, NSP1F, NSP1R2, NSP2F, NSP2R, NSP3F, NSP3R, NSP4F, NSP4R, NSP5F, NSP5R, NSP6F, NSP6R, NSP7F, NSP7R, NSP8F, NSP8R, NSP9R, NSP10F, and T21R (see supplementary Tables S1 and S2 in reference 8). The amplicons were sequenced with an ABI genetic analyzer (Applied Biosystems, USA) and assembled into a near-complete genome using SeqMan version 7.0 (DNAStar Lasergene, USA). Phylogenetic analysis using the partial VP1 region (nucleotides 46 to 633) was carried out in MEGA7 (9).

**FIG 1:**
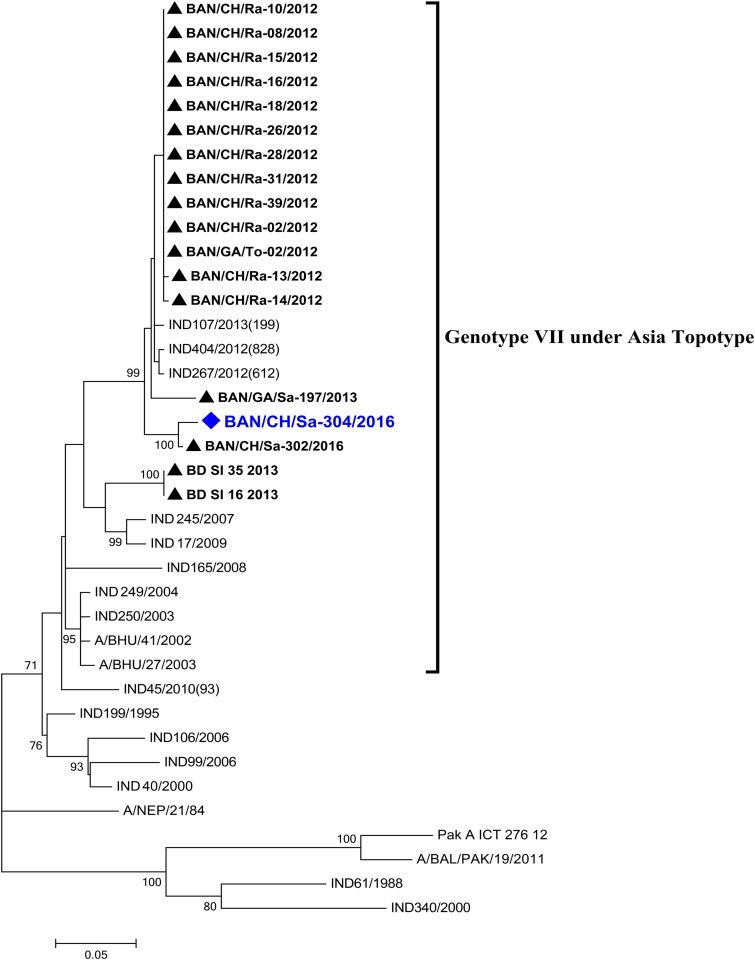
FMDV serotype A partial VP1 coding region-based phylogenetic tree. The tree was reconstructed with the maximum likelihood (ML) method using the Kimura 2-parameter model with 1,000 bootstrap replicates in MEGA7. Phylogeny showed that the vaccine strain, BAN/CH/Sa-304/2016 (indicated by a blue diamond) clustered within genotype VII strains in the Asia topotype along with other Bangladeshi strains (▲).

The near-complete genome of the vaccine candidate BAN/CH/Sa-304/2016 is 8,221 nucleotides (nt) in length, consisting of 53.49% GC content, with a 5′ untranslated region (5′ UTR), coding sequence (CDS), and 3′-UTR region of 1,101, 6,999, and 121 nt, respectively. The CDS codes for a polyprotein 2,332 amino acids (aa) long, containing four structural proteins, VP1 to -4, and eight nonstructural proteins (L, 2A, 2B, 2C, 3A, 3B, 3C, and 3D), while the 5′-UTR poly(C) tract and 3′-UTR poly(A) tail are 16 nt and 25 nt long, respectively. Phylogenetically, the isolates BAN/CH/Sa-304/2016 and BAN/CH/Sa-302/2016 (detected at the same farm on the same day; our unpublished data) clustered in a separate clade closely related to other isolates from Bangladesh and India within genotype VII of the Asia topotype (Fig. 1).

The homology search in GenBank using BLAST showed that BAN/CH/Sa-304/2016 shares 96% nucleotide similarity with previous serotype A isolate BAN/GA/Sa-197/2013 (GenBank accession no. KJ754939) and 91% with Indian vaccine strain IND40/00 (GenBank accession no. HM854025). The VP1, VP2, and VP3 protein sequences of BAN/CH/Sa-304/2016 had 8%, 6%, and 5% variability, with 16, 12, and 9 aa substitutions compared to the respective regions of vaccine strain IND40/00. Only 4 aa substitutions (S83E, T143A, I154V, and D170T) of VP1 were in antigenically critical sites (10). One amino acid deletion was found in the VP3^59^ position of BAN/CH/Sa-304/2016.

We report here the near-complete genome sequence of FMDV serotype A potential vaccine strain BAN/CH/Sa-304/2016 for FMD control in Bangladesh.

### Data availability.

The near-complete genome sequence of FMDV serotype A strain BAN/CH/Sa-304/2016 has been deposited in the NCBI GenBank database under the accession no. MK088171.
